# Effectiveness of Intracervical Foley Catheter for Induction of Labor in Women With Singleton Term Pregnancy and Previous Lower Segment Cesarean Section: A Pilot Study

**DOI:** 10.7759/cureus.78404

**Published:** 2025-02-03

**Authors:** Queena S Dsouza, Sulu E Sasidharan, Teena S Dsouza, Qamariya Ambusaidi, Ashma D Monteiro

**Affiliations:** 1 Department of Obstetrics and Gynecology, Nizwa Hospital, Ministry of Health, Nizwa, OMN; 2 Department of Conservative Dentistry and Endodontics, AB Shetty Memorial Institute of Dental Sciences, NITTE (Deemed to be University), Mangaluru, IND; 3 Department of Data Science, Prasanna School of Public Health, Manipal Academy of Higher Education, Udupi, IND

**Keywords:** induction of labor, intracervical foley catheter, modified bishop score, previous lscs, vbac

## Abstract

Introduction and aim: Current clinical practice advocates and supports a trial of labor after one previous lower segment cesarean section (LSCS) if the pregnant woman wishes to have a vaginal delivery. Induction of labor with pharmacological agents can pose a risk of uterine rupture in women with previous LSCS. Induction with mechanical methods is a safe alternative. However, the data regarding its effectiveness is limited. The present study aimed to assess the effectiveness of intracervical Foley catheter for induction of labor in women with singleton term pregnancy and one previous LSCS.

Materials and methods: This was a cross-sectional retrospective pilot study conducted at the Department of Obstetrics and Gynecology, Nizwa Hospital, Ministry of Health, in Nizwa, Oman. The data of women who fulfilled the eligibility criteria was collected from the hospital's electronic medical records system "Al-Shifa" from October 1, 2023, to March 31, 2024. The change in modified Bishop score, oxytocin requirement, induction delivery interval, mode of delivery, maternal complications, and neonatal outcome were observed. Data analysis was done using the R software (R Foundation for Statistical Computing, Vienna, Austria (https://www.R-project.org/)).

Results: Out of the 64 women who underwent induction of labor, 42 (65.62%) had a vaginal birth after cesarean section (VBAC). There was a statistically significant improvement in the modified Bishop score (p<0.0001) after induction of labor. The VBAC success rate was 84.6% among those with previous VBAC as compared to 52.6% for those without previous VBAC. No significant maternal or fetal complications were observed.

Conclusion: Induction of labor with intracervical Foley catheter serves as an effective method for improving the modified Bishop score in women with previous LSCS and thus increases the chance of a successful VBAC.

## Introduction

Cesarean deliveries are increasingly becoming common worldwide. The World Health Organization and the United Nations Children's Fund have recommended that cesarean section (CS) rates should account for a maximum of 15% of the predicted births [1]. Repeat CS after a previous CS has been a significant contributor to the overall increased CS rate and accounts for more than one-third of all cesarean deliveries all around the world [2].

Fear and unawareness regarding the safety of methods of induction of labor are some of the factors that influence women's decisions on the mode of birth after a previous CS [3], whereas fear of liability is the major reason why obstetricians fear vaginal birth after cesarean section (VBAC) [4].

Induction of labor is a common intervention in obstetrics, with the goal of stimulating uterine contractions before spontaneous labor begins to facilitate a safe vaginal delivery. While induction is generally straightforward in low-risk pregnancies, it presents unique challenges in women with a history of CS. VBAC is often desired to avoid the risks associated with repeat cesarean deliveries, including complications such as placenta accreta, surgical complications, and longer recovery times. However, labor induction in women with a prior lower segment cesarean section (LSCS) carries an inherent risk of uterine rupture, which can result in life-threatening maternal and neonatal outcomes.

Traditionally, labor induction has been performed using pharmacological agents such as prostaglandins and oxytocin to ripen the cervix and stimulate contractions. However, these agents are associated with increased uterine hyperstimulation, particularly in women with a scarred uterus, raising the risk of uterine rupture. Mechanical methods, including the use of an intracervical Foley catheter, have emerged as safer alternatives due to their lower risk of uterine overstimulation. The Foley catheter promotes cervical ripening through mechanical dilation rather than pharmacological action, thereby reducing the stress on the uterine muscle and potentially lowering the risk of adverse events in women attempting VBAC.

Advocating and encouraging the use of mechanical methods of induction of labor like intracervical Foley catheter may help increase the acceptability of trial of labor after cesarean section (TOLAC) and increase the rates of VBAC.

Aim

Although the Foley catheter is widely used as a mechanical method for labor induction, there is a need for more focused research on its effectiveness and safety specifically in women with a history of LSCS. The success of VBAC largely depends on effective cervical ripening and labor progression without compromising the integrity of the uterine scar. The present study aimed to assess the effectiveness of intracervical Foley catheter for induction of labor in women with singleton term pregnancy and one previous LSCS.

## Materials and methods

This pilot study was designed as a cross-sectional retrospective study. It was conducted at the Department of Obstetrics and Gynecology, Nizwa Hospital, Ministry of Health, in Nizwa, Oman. Approval was obtained from the Research and Ethical Review and Approve Committee, Al Dakhiliyah Governorate, Ministry of Health (approval number: 28661). The data of women who fulfilled the eligibility criteria was collected from the hospital's electronic medical records system "Al-Shifa" from October 1, 2023, to March 31, 2024 (over a period of six months).

Participants and procedures

The study population included women who met the following inclusion and exclusion criteria. Inclusion criteria included singleton pregnancy, term gestation (37-41 weeks), one previous LSCS, unfavorable cervix (modified Bishop score ≤6), previous LSCS done for a non-recurring indication, absence of contraindications for VBAC, such as placenta previa or transverse fetal lie, and willingness to attempt VBAC and provide informed consent. In contrast, exclusion criteria included a history of more than one CS, classical or T-shaped uterine scar, significant medical or obstetric conditions contraindicating VBAC (e.g., preeclampsia requiring urgent delivery or uterine rupture in a previous pregnancy), and active labor at the time of admission.

Eligible participants for induction of labor were recruited from the antenatal clinic or the labor and delivery unit. Written informed consent for induction of labor was obtained from all participants. All participants underwent labor induction using an intracervical Foley catheter, which was inserted under sterile conditions. The procedure was performed as follows:

The modified Bishop score was first assessed and recorded. A 20-French Foley catheter was introduced through the external cervical os and advanced until the balloon was located just above the internal os. The balloon was inflated with 60 mL of sterile saline solution. The catheter was secured to the inner thigh with gentle traction. The catheter remained in situ for a maximum of 48 hours or until spontaneous expulsion occurred or active labor ensued. After the expulsion or removal of the intracervical Foley catheter, the modified Bishop score was assessed again, and the participants underwent additional induction methods such as artificial rupture of membranes followed by oxytocin for the augmentation of labor as and when required.

The data regarding the change in modified Bishop score, oxytocin requirement, induction delivery interval, mode of delivery, maternal complications, and neonatal outcome were noted.

Data collection and analysis

The data was collected from the hospital's electronic medical records system "Al-Shifa". The data analysis was performed using the R software (R Foundation for Statistical Computing, Vienna, Austria (https://www.R-project.org/)). Continuous variables were expressed as means with standard deviations, while categorical variables were presented as frequencies and percentages. The Wilcoxon test was used to compare the change in Bishop score before and after induction of labor with intracervical Foley catheter. The chi-squared test was used to find out the association between the previous method of delivery and the present mode of delivery. A p-value of <0.05 was considered statistically significant.

## Results

During the study period, out of the 64 women who underwent induction of labor, 42 (65.62%) had a VBAC, and 22 (34.37%) underwent repeat LSCS. Out of the 42 vaginal deliveries, four women had vacuum-assisted vaginal delivery. The mean age of the participants was 33.8±5.17. The majority of the study population (n=43; 67.2%) were multiparous. The mean gestational age was 38.45±1.08. Majority of the women (n=44; 68.8%) had a BMI of <30. The most common indication for induction of labor was oligohydramnios (n=24; 37.5%).

There was a statistically significant difference seen between the two outcome variables (LSCS and VBAC) in terms of age, number of previous VBAC, and number of days of hospital stay (Table 1).

**Table 1:** Descriptive statistics with respect to mode of delivery (VBAC and LSCS) At a 5% level of significance, the p-value is reported for the two-sample t-test (two-sided) under the null hypothesis of no difference in mean between the two groups for the age variable. For the rest of the variables, the Mann-Whitney U test is conducted, and the p-value is reported at a 5% level of significance under the null hypothesis of no difference in median between the two groups. VBAC: vaginal birth after cesarean section; LSCS: lower segment cesarean section

	LSCS (n=22)	VBAC (n=42)	P-value
	Mean±SD	Mean±SD	
Age	31.5±4.52	35.1±5.11	0.008
No. of previous VBAC	0.27±0.63	1.09±1.39	0.007
No. of days of hospital stay	3.86±0.77	2.48±0.51	<0.001

In women who had a VBAC after induction of labor, 15 women (35.71 %) required augmentation of labor with oxytocin, and 30 women (71.42%) delivered within 24 hours of induction of labor.

The present study showed that there was a significant change in the modified Bishop score after induction of labor with intracervical Foley catheter (Table 2).

**Table 2:** Modified Bishop score before and after induction of labor Wilcoxon signed-rank test: At a 5% level of significance, we conclude that there is a significant difference in Bishop score before and after induction of labor (p<0.001).

Modified Bishop score	Mean±SD	P-value
Pre-induction	2.83±0.58	<0.001
Post-induction	6.36±1.47	

The VBAC success rate was 84.6% among those with previous VBAC as compared to 52.6% for those without previous VBAC (Table 3).

**Table 3:** Comparison of the present mode of delivery and the previous most recent mode of delivery Chi-squared test of association: At a 5% level of significance, we conclude that there is a significant association between the previous method of delivery and the outcome variable (LSCS or VBAC). VBAC: vaginal birth after cesarean section; LSCS: lower segment cesarean section

Previous most recent mode of delivery	Present mode of delivery			P-value
	LSCS	VBAC	Total	
LSCS	18 (47.4%)	20 (52.6%)	38 (100%)	0.008
VBAC	4 (15.4%)	22 (84.6%)	26 (100%)	
Total	22 (34.4%)	42 (65.6%)	64 (100%)	

The most important indication for emergency LSCS was fetal distress (59.10%) as evidenced by cardiotocography (CTG) abnormalities.

There were no significant maternal or neonatal complications noted except for one case of postpartum fever and two cases of neonatal respiratory distress. No maternal or neonatal deaths were seen (Table 4).

**Table 4:** Maternal and neonatal morbidity VBAC: vaginal birth after cesarean section; LSCS: lower segment cesarean section

	LSCS (n=22)	VBAC (n=42)
Maternal postpartum fever	1 (4.54%)	0 (0%)
Neonatal respiratory distress	1 (4.54%)	1 (2.38%)

## Discussion

The use of the intracervical Foley catheter for labor induction, particularly in women with a history of one LSCS, has been explored in several studies, but the findings remain variable. Our results support previous literature indicating that the Foley catheter is a safe and effective method of labor induction in this population.

In our study, there was a statistically significant difference between the mode of delivery in terms of the mean age of women undergoing induction of labor. As per our study, elderly women (>35 years) were more likely to have a successful VBAC when compared to younger women. However, other studies like the one by Srinivas et al. [5]have shown conflicting results where women who were of advanced maternal age (≥35 years) were more likely to experience an unsuccessful trial of labor.

In the present study, there was a statistically significant improvement in the modified Bishop score observed with Foley catheter induction, thereby suggesting that intracervical Foley catheter can be an effective method of induction of labor in women with one previous LSCS with unfavorable cervix. This was comparable with the study by Hemalatha and Swetha, who observed statistically significant improvement in the Bishop score ranging from 2 to 5 at the time of Foley insertion to 4 to 10 at the time of removal [6]. Another study by Jakhar and Malla conducted in India showed similar results [7].

In the current study, the overall success rate of VBAC was 65.62%. This was comparable to a study by Gonsalves et al. where the VBAC success rate was 69.1% [8]. Another study by Jozwiak et al. showed similar results with a VBAC success rate of 71%, with two perinatal deaths (1%), one of which was due to uterine rupture (0.5%) [9].

In our study group, there was no incident of uterine rupture. Studies done by Sananès et al. [10], Hemalatha and Swetha [6], Gonsalves et al. [8], and Ben-Aroya et al. [11] also did not report any case of uterine rupture.

A significant association was seen between the previous method of delivery and the outcome variable (LSCS or VBAC). The VBAC success rate was 84.6% among those with previous VBAC as compared to 52.6% for those without previous VBAC suggesting that the best predictor for a successful VBAC is a previous VBAC. Studies by Srinivas et al. [12]  and Yokoi et al. [13] have shown similar results.

Since this study was a pilot study, the sample size was small. So this limitation may hinder the generalizability of the study results with the general population. Another limitation of the study was that in our institution, intracervical Foley catheter is the only method of induction of labor that is used in patients with one previous LSCS and unfavorable cervix. As a result, we could not compare this method with other methods of induction of labor and compare the success rates and safety profile.

## Conclusions

Our study showed that induction of labor with intracervical Foley catheter serves as an effective method for improving the modified Bishop score in women with previous LSCS. It also influences the mode of delivery in these women and increases the chance of a successful VBAC. In addition, a previous VBAC will increase the likelihood of having a subsequent VBAC.

Current guidelines from organizations such as the American College of Obstetricians and Gynecologists (ACOG) recommend mechanical methods like the Foley catheter as a preferred option for induction of labor in women with a prior CS, especially those attempting VBAC.Our research supports these recommendations, adding new evidence from a specific cohort of women with one prior LSCS. As VBAC continues to be promoted as a means to reduce repeat CS and associated long-term complications (e.g., placental abnormalities in future pregnancies), the Foley catheter's role in facilitating safe labor induction is becoming increasingly important.

In contrast, while oxytocin remains a commonly used agent for induction, studies have shown an increased risk of uterine rupture when used in women with a prior LSCS, particularly when combined with other agents. This highlights the importance of choosing induction methods that prioritize uterine safety while ensuring labor progresses efficiently. The Foley catheter, in this context, remains a valuable tool.

## References

[1] (2009). Geneva: WHO, UNICEF. Monitoring emergency obstetric care: a handbook.

[2] Cheng YW, Eden KB, Marshall N, Pereira L, Caughey AB, Guise JM (2011). Delivery after prior cesarean: maternal morbidity and mortality. Clin Perinatol.

[3] Hamilton EL, McLaughlin K, Mollart L (2023). Factors that influence women's decision on the mode of birth after a previous caesarean section: a meta-ethnography. Int J Community Based Nurs Midwifery.

[4] Cox KJ (2011). Providers' perspectives on the vaginal birth after cesarean guidelines in Florida, United States: a qualitative study. BMC Pregnancy Childbirth.

[5] Srinivas SK, Stamilio DM, Sammel MD, Stevens EJ, Peipert JF, Odibo AO, Macones GA (2007). Vaginal birth after caesarean delivery: does maternal age affect safety and success?. Paediatr Perinat Epidemiol.

[6] Hemalatha KR, Swetha D (2017). Case series of Foley's induction in patients with previous caesarean. Int J Reprod Contracept Obstet Gynecol.

[7] Jakhar S, Malla VG (2020). Use of intracervical Foley catheter for pre-induction cervical ripening in women planned for vaginal birth after previous caesarean section. Int J Reprod Contracept Obstet Gynecol.

[8] Gonsalves H, Al-Riyami N, Al-Dughaishi T, Gowri V, Al-Azri M, Salahuddin A (2016). Use of intracervical Foley catheter for induction of labour in cases of previous caesarean section: experience of a single tertiary centre in Oman. Sultan Qaboos Univ Med J.

[9] Jozwiak M, van de Lest HA, Burger NB, Dijksterhuis MG, De Leeuw JW (2014). Cervical ripening with Foley catheter for induction of labor after cesarean section: a cohort study. Acta Obstet Gynecol Scand.

[10] Sananès N, Rodriguez M, Stora C (2014). Efficacy and safety of labour induction in patients with a single previous caesarean section: a proposal for a clinical protocol. Arch Gynecol Obstet.

[11] Ben-Aroya Z, Hallak M, Segal D, Friger M, Katz M, Mazor M (2002). Ripening of the uterine cervix in a post-cesarean parturient: prostaglandin E2 versus Foley catheter. J Matern Fetal Neonatal Med.

[12] Srinivas SK, Stamilio DM, Stevens EJ, Odibo AO, Peipert JF, Macones GA (2007). Predicting failure of a vaginal birth attempt after cesarean delivery. Obstet Gynecol.

[13] Yokoi A, Ishikawa K, Miyazaki K, Yoshida K, Furuhashi M, Tamakoshi K (2012). Validation of the prediction model for success of vaginal birth after cesarean delivery in Japanese women. Int J Med Sci.

